# Tumour-associated glial host cells display a stem-like phenotype with a distinct gene expression profile and promote growth of GBM xenografts

**DOI:** 10.1186/s12885-017-3109-8

**Published:** 2017-02-07

**Authors:** Lina Leiss, Ercan Mutlu, Anne Øyan, Tao Yan, Oleg Tsinkalovsky, Linda Sleire, Kjell Petersen, Mohummad Aminur Rahman, Mireille Johannessen, Sidhartha S. Mitra, Hege K. Jacobsen, Krishna M. Talasila, Hrvoje Miletic, Inge Jonassen, Xingang Li, Nicolaas H. Brons, Karl-Henning Kalland, Jian Wang, Per Øyvind Enger

**Affiliations:** 1 Neuro Clinic, Haukeland University Hospital, Bergen, Norway; 2 Oncomatrix Research Lab, Department of Biomedicine, University of Bergen, Bergen, Norway; 3 Department of Clinical Science, University of Bergen, Bergen, Norway; 4 Department of Microbiology and Immunology, Haukeland University Hospital, Bergen, Norway; 5 Department of Neurosurgery, Qilu Hospital of Shandong University, Jinan, China; 6 Brain Science Research Institute, Shandong University, 107# Wenhua Xi Road, Jinan, 250012 People’s Republic of China; 7 Computational Biology Unit, Uni Computing, Uni Research AS, Bergen, Norway; 8 Institute for Stem Cell Biology and Regenerative Medicine, Stanford University, Stanford, California, USA; 9 Translational Cancer Research Group, Department of Biomedicine, University of Bergen, Bergen, Norway; 10 Department of Clinical Medicine, Haukeland University Hospital, Bergen, Norway; 11 Department of Informatics, University of Bergen, Bergen, Norway; 12 Core Facility Flow Cytometry, Centre de Recherche Public de la Santé (CRP-Santé), L-1526 Luxembourg, Luxembourg; 13 Department of Neurosurgery, Haukeland University Hospital, Bergen, Norway

**Keywords:** Glioblastoma, Tumour-host interplay, Tumour-associated glial cells, GFF-NOD/scid mice, Xenograft tumours, Gene expression analysis, Stem cell markers, POU3F2

## Abstract

**Background:**

Little is known about the role of glial host cells in brain tumours. However, supporting stromal cells have been shown to foster tumour growth in other cancers.

**Methods:**

We isolated stromal cells from patient-derived glioblastoma (GBM) xenografts established in GFP-NOD/scid mice. With simultaneous removal of CD11b^+^ immune and CD31^+^ endothelial cells by fluorescence activated cell sorting (FACS), we obtained a population of tumour-associated glial cells, TAGs, expressing markers of terminally differentiaed glial cell types or glial progenitors. This cell population was subsequently characterised using gene expression analyses and immunocytochemistry. Furthermore, sphere formation was assessed in vitro and their glioma growth-promoting ability was examined in vivo. Finally, the expression of TAG related markers was validated in human GBMs.

**Results:**

TAGs were highly enriched for the expression of glial cell proteins including GFAP and myelin basic protein (MBP), and immature markers such as Nestin and O4. A fraction of TAGs displayed sphere formation in stem cell medium. Moreover, TAGs promoted brain tumour growth in vivo when co-implanted with glioma cells, compared to implanting only glioma cells, or glioma cells and unconditioned glial cells from mice without tumours. Genome-wide microarray analysis of TAGs showed an expression profile distinct from glial cells from healthy mice brains. Notably, TAGs upregulated genes associated with immature cell types and self-renewal, including *Pou3f2* and *Sox2*. In addition, TAGs from highly angiogenic tumours showed upregulation of angiogenic factors, including *Vegf* and *Angiopoietin 2*. Immunohistochemistry of three GBMs, two patient biopsies and one GBM xenograft, confirmed that the expression of these genes was mainly confined to TAGs in the tumour bed. Furthermore, their expression profiles displayed a significant overlap with gene clusters defining prognostic subclasses of human GBMs.

**Conclusions:**

Our data demonstrate that glial host cells in brain tumours are functionally distinct from glial cells of healthy mice brains. Furthermore, TAGs display a gene expression profile with enrichment for genes related to stem cells, immature cell types and developmental processes. Future studies are needed to delineate the biological mechanisms regulating the brain tumour-host interplay.

**Electronic supplementary material:**

The online version of this article (doi:10.1186/s12885-017-3109-8) contains supplementary material, which is available to authorized users.

## Background

Glioblastomas (GBMs) are aggressive brain tumours, characterised by angiogenesis and infiltrative growth [1]. They have a poor prognosis with virtually no long time survivors, and new therapies are urgently needed. Although glioma stem cells have been implicated in brain tumourigenesis [2, 3], conflicting data suggest that additional mechanisms are involved [4, 5].

In the non-pathological state, the roles of glial cells in the CNS overlap with functions of fibroblasts in other organs, such as secretion of ECM components, provision of structural support and homeostasis. Astrocytes can adapt a spectrum of altered phenotypes [6] in health and disease, in response to changing external cues. They may regulate normal brain function by modulating transmission in glial and neuronal cell signalling networks [6]. Activation of astrocytes during trauma and inflammation is characterised by proliferation, increased expression of GFAP and release of cytokines and neurotrophic factors [7]. Numerous studies show that fibroblasts in the tumour microenvironment undergo activation to promote cancer growth [8–10]. This activated state is characterised by cell proliferation, release of growth factors and matrix metalloproteinases [11]. However, fewer studies have so far addressed whether glial cells and fibroblasts have similar roles in tumour progression.

We previously reported that astrocytes can modulate the chemosensitivity of glioma cells to clinically relevant drugs using a tumour-stroma co-culture model with glioma cell-specific luminescence [12]. Recently, Sin et al. [13] reported that astrocytes promoted invasion of the GL261 glioma cell line in mice. Moreover, other glial cell types such as oligodendrocyte progenitor cells have been shown to promote neovascularisation in gliomas [14]. Thus, several studies suggest that heterotypic signalling circuits involving glial host cells and glioma cells are integral parts of tumour progression. Previously, tumour-associated astrocytes were studied in heterozygously deleted *Ink4a/Arf* mice with PDGF-induced murine gliomas. Notably, these transgenic tumour-associated astrocytes displayed a gene expression profile distinct from normal astrocytes, suggesting a role in antigen presentation [15]. However, these astrocytes carried a tumour suppressor deletion that may limit the relevance of these findings to the microenvironment of human glioma cells. Thus, little data are available regarding how glial cells in the tumour microenvironment are reprogrammed during brain tumour progression and how this impacts on overall disease course.

Investigating the role of tumour-associated glial cells (TAGs) in malignant brain tumours is challenging since no markers reliably distinguish reactive glial cells from neoplastic glioma cells [16]. Additionally, glial cells are phenotypically diverse [17] and cannot be identified by any unifying marker. Previously, we established brain tumours in nude rats with non-angiogenic and vascular, mature GBM phenotypes using human GBM biopsies [18, 19]. The non-angiogenic phenotype displays infiltrative growth and atypia similar to GBMs, but with little or no angiogenesis. The vascular, mature phenotype also displays angiogenesis. In order to investigate the roles of TAGs, we established these tumours in GFP-NOD/scid mice [20], resulting in GFP^+^ host cells from two different tumour phenotypes and GFP^−^ tumour cells. TAGs were obtained by FACS isolation of GFP^+^ cells, with simultaneous removal of cells expressing vascular or immune cell surface markers CD31 and CD11b, respectively. Since the onset of angiogenesis is considered a key event in gliomas, coinciding with worsening of the prognosis [21], we isolated TAGs from both the non-angiogenic and the mature vascular GBM tumour phenotypes. We then investigated their functional properties, and conducted gene expression profiling of these TAGs that was subsequently validated in human GBMs.

## Methods

### Cell culture

Biopsies were obtained with written consents of the patients from the Department of Neurosurgery, Haukeland University Hospital, Bergen, Norway. Collection of tumour biopsies was approved by the Regional Ethical Committee (REK Vest). Biopsy spheroids were prepared as previously described, and the resulting spheroids have previously been shown to contain both glioma cells as well as stromal elements from the brain [22]. In brief, tissue samples were minced into 0.5 mm^3^ fragments and placed into agar-coated tissue culture flasks with complete DMEM; DMEM culture medium (Sigma-Aldrich, St. Louis, MO, USA) containing 10% fetal bovine serum (FBS) supplemented with NEAA, 100 U/ml Pen/Strep and 400 μM L-glutamine, all from Cambrex (Cambrex, East Rutherford, NJ, US). Biopsy spheroids were maintained in a standard tissue culture incubator with 5% CO_2_ in air and 100% relative humidity at 37 °C and the medium was changed once a week.

### Animal experiments

Tumour xenografts were established as previously described [18], In short, human GBM biopsy spheroids of 250 μm in diameter were selected after 1–2 weeks in culture, using a microscope (Olympus CKX31, Olympus Microscopy, Essex, UK) with a reticular eye piece. 10 biopsy spheroids were implanted in each GFP-NOD/scid mouse 1.5 mm to the right of the midline, 1 mm posterior to the bregma suture and 2 mm below the cortical surface. In experiments not involving FACS sorting, we used NOD/scid mice (GFP negative). Marcain was injected in the scalp and the mice were operated under isoflurane gas anaesthesia, immobilised in a stereotactic frame (Model 900, David Kopf Instruments, Tujunga USA). In the co-implantation experiments, we implanted cell suspensions in PBS following the same operative procedure. The cell suspensions contained 50,000 tumour cells, mixtures of 50,000 tumour and 50,000 TAGs or normal glial cells, and controls containing 50,000 TAGs only. In total we used 58 mice for establishing the tumour phenotypes in vivo, and the co-implantation experiments. The mice used for isolation of TAGs and normal glial cells were age-matched, fully adult mice from both genders, 3–4 months old. The National Animal Research Authority in Norway approved the experiments, and the animals were kept in an isolation facility at 25 °C (55% relative humidity) in a specific pathogen free environment. They were fed a standard pellet diet and provided water ad libitum.

### MR imaging

MR images were acquired using a 7 T small-animal MR scanner (Pharmascan, Bruker Biospin, Billerica, MA, USA) as previously described [23]. In short, animals were anesthetised using 1–2% isoflurane mixed with equal parts N_2_ and O_2_ supplied via a mask. Animals were placed in a prone position in a cradle containing a heating pad at 37 °C. Respiration was monitored throughout the experiment (SA Instruments Inc., NY, USA).

MRI protocols were as follows: MR sequences included T2-wegithed RARE sequence with TR/TE of 4200/35.2 ms, and T1-weighted RARE sequence before and after subcutaneous injection of contrast agent, 0.1 ml of 0.5 mmol/ml Omniscan (Nycomed Amersham, Oslo, Norway), with TR/TE 1000/9.0 ms. T1 and T2 weighted sequences had FOV of 2.0 × 2.0 cm and a matrix size of 256 × 256. 12 slices, each 1 mm thick were collected in all sequences.

### Tumour dissociation

To flush out blood cells the mice were perfused with saline buffer with heparin, before the brains were dissociated. GBM xenografts and healthy brains from GFP-NOD/scid mice were minced with scalpels, followed by dissociation with 1 mg/ml collagenase/dispase (Roche, Rotkreuz, Switzerland) and 0.125% DNase I (Sigma-Aldrich) dissolved in complete DMEM for 60–90 min at 37 °C. The dissociated tissue was washed with ice cold HBSS and filtrated twice through a 70 μm cell strainer (Millipore, Billerica, MA, USA). The cell suspension was centrifuged on 300 g at 4 °C for 10 min. Pellets were resuspended in PBS with 2% FBS.

### FACS sorting

After dissociating the GBM xenografts, cells were stained with anti-mouse CD31- APC and CD11b- APC antibodies (1:100, eBioscience, San Diego, CA, USA) on ice in the dark for 15 min, followed by washing twice with ice cold FACS buffer (PBS with 1% FBS). Cells were filtered through a 40 μm cell strainer (Millipore) to obtain a single cell suspension. GFP-negative and -positive cells were analysed and separated using a cell sorter (FACS Aria SORP, BD Biosciences, Erembodegem, Belgium). Separation was based on gating for single cells (FSC-W/FSC-A), viability by Sytox blue (LifeTechnologies, Carlsbad, CA, USA) exclusion and GFP fluorescence. From the GBM xenografts, endothelial cells and immune cells recognised by APC-conjugated anti-CD31 and anti-CD11b were gated out. We performed control fluorescence microscopy of the cells collected from the sorting procedure for confirming purity. In order to obtain normal glial cells as controls for cell-cycle, FCM analysis of marker expression, sphere formation and co-implantation studies we used the same dissociation protocol for healthy mouse brains followed by FACS isolation with exclusion of CD11b^+^ and CD31^+^ cells. Due to the high lipid content of normal mouse brains, FACS sorting with exclusion of CD11b and CD31 from these samples was omitted as we were unable to obtain sufficient amounts of RNA when FACS sorting. Thus, we instead validated the gene expression analysis data extensively performing IHC of both mouse brains, which confirmed the upregulation of markers as suggested by the gene expression analysis (below).

### Spheroid formation assay

Stromal cells isolated from GBM xenograft tumours and from healthy mice brains were separately resuspended in Neurobasal stem cell medium (Invitrogen, Carlsbad, CA, USA) supplemented with 1xB-27 (without Vitamin A), 1xGlutaMax I (Invitrogen), 20 ng/ml EGF (Sigma-Aldrich), 20 ng/ml FGF2 (R&D Systems, Minneapolis, MN) and 100 U/ml Pen/Strep (Cambrex). 1500 cells were seeded in each well of a 6-well plate for each cell type. The cell cultures were inspected daily, and sphere formation (minimum 50 cells in a spheroid) was assessed after 9 days, by manual counting under an inverted microscope (Olympus CKX31). Bright field images were obtained with Nikon TE2000-E (Nikon Instruments Inc., Melville, NY, USA) using the NIS Elements Software (Nikon Instruments Inc.).

### RNA isolation

Total RNA was extracted using RNEasy Mini Kit (Qiagen, Hilden, Germany). Briefly, the sorted cells were collected by centrifugation and dissolved in RLT lysis buffer. The remaining procedure was performed according to the manufacturer’s instructions including treatment with DNase I (Qiagen).

### Gene expression analysis

The global mRNA expression of each sample was measured using the Agilent Whole Mouse Genome 4×44K Oligo Microarray with Sure Print Technology (Agilent, Palo Alto, USA). 1 μg of DNase-treated total RNA was converted into cDNA and Cy3-labeled cRNA using the Low RNA Input Linear Amplification Kit PLUS, One-Color kit (Agilent, Santa Clara, CA, USA). Microarrays were scanned with an Agilent scanner G2505B bundle and images were analysed using Agilent Feature Extraction Software v.9.1. After background correction and normalisation, the resulting raw data files were imported into the J-Express analysis suite [24] for preprocessing and gene expression analysis. The gMeanSignal was used as signal for each probe. Control probes were filtered out and duplicate probes were combined by taking the median signal of the probes. Signal intensities were quantile normalised [25] to achieve an inter-array normalised gene expression data matrix. Finally, the data was log_2_ transformed and multiple probes for the same Gene Symbol (Agilent annotation file 014868) were combined using the MaxProbe statistics [26].

### Immunocytochemistry and BrdU pulsing

Cells were fixed with 4% paraformaldehyde for 10 min, and permeabilized for 4 min with 0.5% Triton X-100 in PBS. Blocking was done using 0.5% BSA in PBS for 15 min. All steps were performed in room temperature. Mice were pulsed intraperitoneally with BrdU (150 mg/kg) 45 min prior to harvesting and dissociating the brains. Cells were incubated with mouse anti-BrdU (Abcam, Cambridge, MA, USA) at 1:100, containing 3 μl (1U/μl) DNase I (Qiagen) for 45 min at 37 °C. Following incubation, cells were washed in PBS and incubated with secondary antibody (diluted 1:100 in blocking buffer) for 45 min at 37 °C. The secondary antibody was TXRD-conjugated goat anti-mouse (Southern Biotech, Birmingham, AL, USA). After washing with PBS cells were mounted with Vectashield mounting medium containing DAPI (Vector Laboratories, Burlingame, CA, USA). Fluorescent images were obtained with a Zeiss LSM 510 Meta confocal microscope (Carl Zeiss MicroImaging, Jena, Germany), using a 63× oil immersion objective.

### Immunohistochemistry

Mouse brains were embedded in Tissue-Tek O.C.T. (Sakura Finetek, Alphen aan den Rijn, The Netherlands) and snap frozen in isopentane (Sigma-Aldrich) cooled on dry ice. Snap-frozen tissue was sectioned at 6 μm thickness on a cryostat (Leica CM3050S, Leica Microsystems, Wetzlar, Germany). Subsequent washes were done with TBS-Tween20 (Sigma-Aldrich) wash buffer, 3×3 min, all steps were performed at room temperature. The primary antibodies used were: anti-ANGPT2 (1:100, Abcam), anti-Msi1 (1:500, Abcam), anti-NG2 (1:100, Abcam), anti-Sox2 (1:100, Abcam), anti-VEGF (1:20, Abcam), anti-Vimentin (1:100, Abcam), anti-PDGFRα (1:100, Cell Signaling Technology, Beverly, MA, USA), anti-GFAP (1:500, Dako, Glostrup, Denmark), anti-Tubulin β3 (1:100, Millipore), anti-HuNu (1:100, Millipore), anti-FGF2 (1:100, Santa Cruz Biotechnology, Santa Cruz, CA, USA), anti-IDH1 (Dianova, Hamburg, Germany) and anti-POU3F2 (SC-2895, Santa Cruz). The secondary antibodies used were: FITC-conjugated goat anti-rabbit (1:200, Southern Biotech), FITC-conjugated goat anti-mouse (1:200, Southern Biotech), TXRD-conjugated goat anti-mouse (1:100, Southern Biotech).

### Flow cytometry

TAGs and normal glial cells acutely isolated by FACS were fixed in 4% PFA (Sigma-Aldrich) for 10 min, pelleted and permeabilized with 0.5% Triton X-100 (Sigma-Aldrich) for 4 min. Samples were pelleted again and blocked in 0.5% BSA (Sigma-Aldrich) for 15 min before immunostaining. Antibodies were diluted to 1:100; anti-GFAP (Dako), anti-Nestin (Abcam), anti-beta-tubulin III (Abcam), anti-MBP (Millipore) and 1:10; anti-O4 (RnD Systems) in 100 μl blocking buffer, and samples were incubated in 100 μl staining reaction for one hour at room temperature. Samples were washed in 3 ml blocking buffer, stained with Alexa Fluor 647-conjugated secondary anti-mouse and anti-rabbit antibodies (both from LifeTechnologies) in 100 μl for 45 min at room temperature and washed in 3 ml blocking buffer. Samples were resuspended in 100 μl 1xPBS before analysis, and cells stained with secondary antibodies alone or IgM isotype control (for O4, BD Biosciences) were used to set the gates. For cell cycle analysis, acutely isolated TAGs and glial cells from normal mice brains were fixed in 100% ice cold ethanol for 20 min, washed in 1xPBS and incubated for 30 min in RNase (1 mg/ml, Sigma-Aldrich) and propidium iodide (50 mg/ml, Sigma-Aldrich). All staining was acquired on AccuriC6 (BD Biosciences) and FlowJo (FlowJo, LCC, Oregon, USA) was used for analysis.

### Statistics

Sphere formation was analysed using Student’s *t*-test with a 2-tailed distribution analysis. Survival data were analysed using the log-rank test. A *p*-value < 0.05 was considered significant. Differentially expressed genes were identified between pairs of sample groups, utilising unpaired SAM [27] analysis implemented in J-Express [24], with 1000 permutations, and considering the list of genes with q < 0.05 as significantly differentially expressed.

Gene Ontology overrepresentation analysis was performed in J-Express software using a Fisher exact test to identify GO terms overrepresented among differentially expressed genes compared to the full data set. Functional term analysis was performed using the DAVID bioinformatics resource [28], with the Agilent Probe IDs as the query. Gene Ontology (GO) terms were considered enriched if their Benjamini-Hoschberg-adjusted *p*-value was less than 0.05 [29]. The analysis of overlap with the human profile genes was done using a custom R-script in the gplots package (http://cran.r-project.org/web/packages/gplots/). A bootstrapping-based analysis with resampling, corresponding to the number of genes in each of the groups, was performed to estimate expected random overlap (mean/sd). These values were subsequently used to determine the probability (significance) of the observed overlap. Human orthologues were identified using mammalian orthology tables available through the mouse genome informatics database at Jackson Laboratories (http://www.informatics.jax.org/orthology.shtml).

## Results

### FACS isolation of GFP^+^CD11b^−^CD31^−^ cells from gliomas in GFP-NOD/scid mice provides a population highly purified for host cells expressing glial markers

**Fig. 1:**
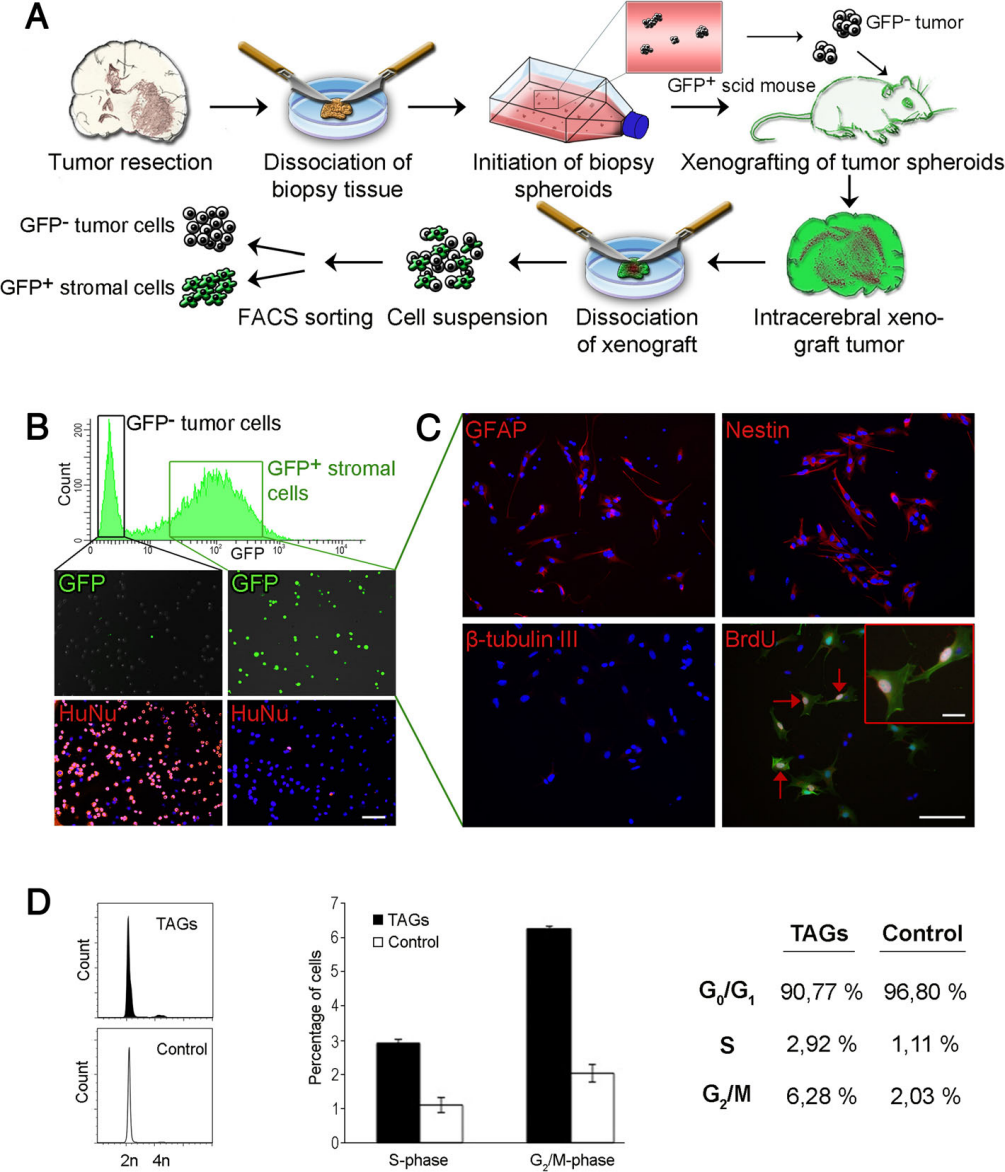
Isolation of tumour-associated glial cells. **a** GBM spheroids established from patient biopsies are implanted in GFP-NOD/scid mice. The resulting tumours are dissociated and GFP+ stromal cells are FACS- sorted with removal of CD31+ and CD11b + cells. **b** Flow cytometry histogram displays a bimodal curve, reflecting a non-fluorescent and a fluorescent cell population (*Upper panel*, Y-axis; cell count, X-axis; GFP fluorescence). Control fluorescence microscopy of cells sorted by GFP expression (*middle panels*) and after staining for human nuclear factor, a pan-human specific marker (HuNu, *lower panels*). **c** ICC for GFAP, Nestin, β-tubulin III and BrdU as indicated. Scale bar: 50 μm, insert: 20 μm. **d** Representative histograms (*left panels*) from cell cycle analysis of acutely isolated TAGs (*n* = 3, *black*) and control cells from normal mouse brain. (*n* = 3, *white*). *Middle panel* shows the percentage differences in S and G_2_/M-phase between TAGs and control, while numbers for all cell cycle phases are outlined in the table (*right*)

The experimental flow-chart is shown in Fig. 1a. We used GFP-NOD/scid mice as a host to establish non-angiogenic and vascular GBM xenografts from patient GBM biopsies [18, 19] (Additional file 1: Figure S1a, b). These tumours were dissociated and flow cytometry histograms showed bi-modal curves, reflecting the presence of fluorescent host cells and non-fluorescent tumour cells (Fig. 1b, top panel). FACS sorting by GFP expression was conducted, with removal of CD31^+^ and CD11b^+^ cells from the host cells (Additional file 1: Figure S1c–e), providing GFP^+^CD31^−^CD11b^−^ cells. In total, we performed FACS of 14 xenograft tumours, including 6 non-angiogenic and 8 mature GBM phenotypes (Additional file 1: Figure S1c–e). The average fraction of GFP^+^CD11b^−^CD31^−^ host cells was 19.1% (12–26.5%, Additional file 1: Figure S1e), being highest in the non-angiogenic tumours. Immunocytochemistry (ICC) and flow cytometry (FCM) analysis demonstrated expression of Nestin, O4, GFAP and myelin basic protein (MBP), whereas expression of the neuronal β-tubulin III was low (Fig. 1c, and Additional file 1: Figure S1f). FCM analysis of 3 GBM xenografts using antibodies against a panel of glial markers confirmed that 96% of these cells expressed GFAP and 90% expressed MBP consistent with co-expression of several glial markers, whereas Nestin and O4 were expressed at somewhat lower rates (Additional file 1: Figure S1f). Thus, GFP^+^CD11b^−^CD31^−^ cells were defined as tumour-associated glial cells (TAGs). Similarly, healthy mice brains (normal brains without tumours) were dissociated and FACS sorted to obtain normal glial cells as controls and their expression of glial markers were subsequently assessed by FCM (Additional file 1: Figure S1f). Notably, a proportion of the TAGs were BrdU-positive (insert, lower right panel Fig. 1c), suggesting that these were actively cycling cells. Cell cycle distribution determined by FCM confirmed that a significantly higher proportion of TAGs were in S or G_2_/M-phase, compared to glial cells from normal brain (Fig. 1d). Control fluorescence microscopy for GFP expression (Fig. 1b, middle panels) as well as immunostaining (Fig. 1b, lower panels) for a pan-human specific nuclear antigen HuNu [30] confirmed high purity of the host cell populations (>98%), with few tumour cells present. The purity was also confirmed by implanting pure TAG cell suspensions, which did not produce tumours (see later section).

### Tumour-associated glial cells form spheres in stem cell medium and promote brain tumour growth in vivo

**Fig. 2:**
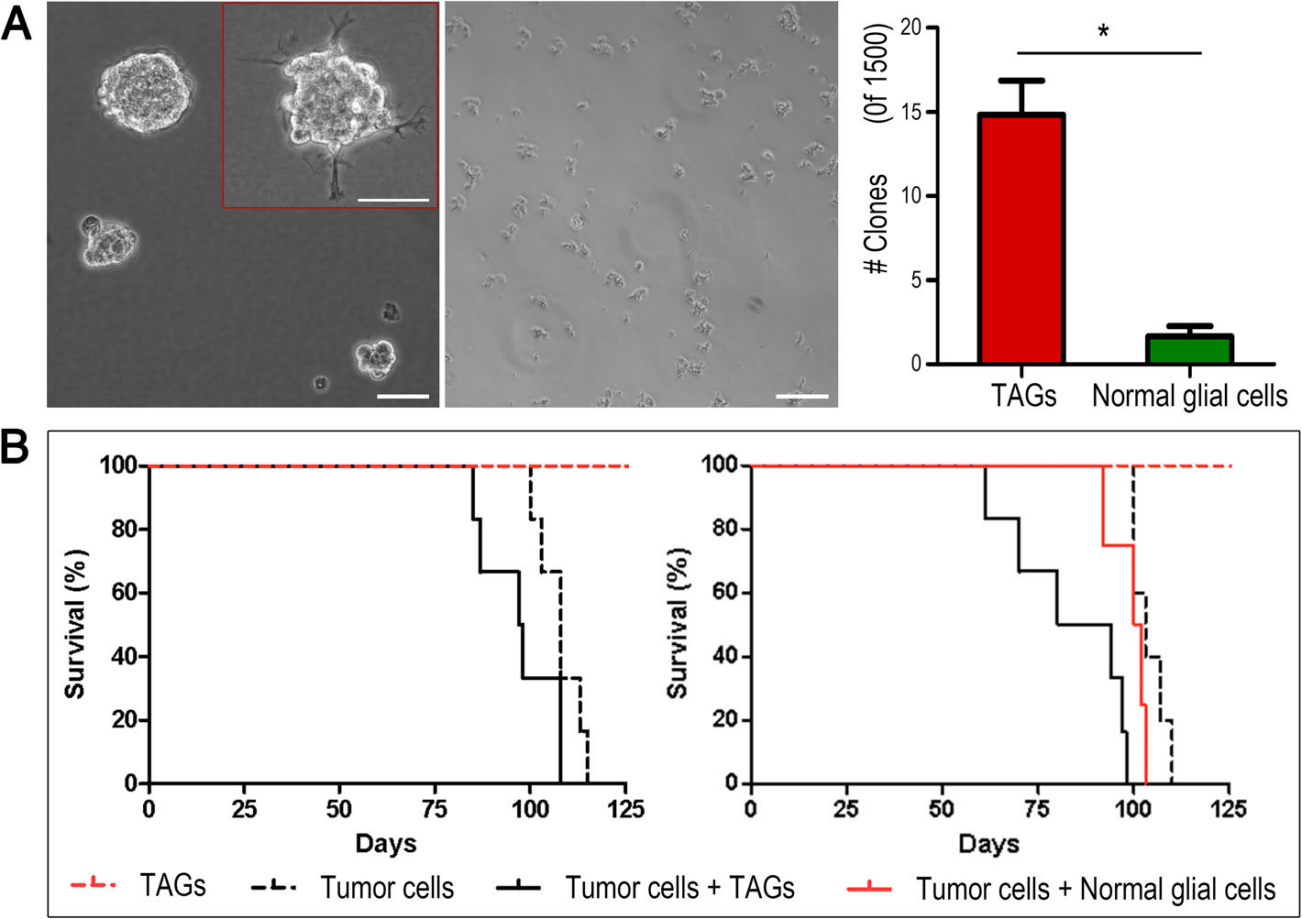
TAGs form spheres in vitro and promote brain tumour growth in vivo. **a** TAGs cultured in stem cell medium formed spheres (*left panel*), whereas normal glial cell sphere formation was barely detectable (*middle panel*), and significantly lower (*right panel*). Scale bar: 100 μm **b** Survival following implantation in mice with the cell suspensions indicated, 50 000 cells of each cell type. Mice were implanted with glioma cells and TAGs from non-angiogenic (*left panel*) and mature GBM (*right panel*) phenotypes

TAGs isolated from mouse brain GBM xenografts were seeded in stem cell medium, and sphere formation was compared with glial cells from healthy mouse brain (Fig. 2a, left and middle panels, respectively). TAGs formed spheres at a frequency of 1%, compared to 0.1% for normal glial cells (*p* = 0.001, Fig. 2a, right panel). In addition, some TAG spheres grew adherent (Fig. 2a, insert left panel). We then assessed whether TAGs promoted brain tumour growth in vivo. First, we isolated TAGs and glioma cells from non-angiogenic xenografts, and re-implanted glioma cells (*n* = 12, 6 in each group), either with TAGs or as pure glioma cell suspensions (Fig. 2b, left panel). In addition, 4 mice received only TAGs, and did not develop tumours. Mice co-implanted with TAGs and glioma cells had a shorter survival (98 days) than mice implanted with glioma cells only (108 days), although the difference was not significant (*p* = 0.062). One mouse receiving only TAGs died after almost 6 months, but histological examination showed no tumour in its brain or in any of the other mice receiving TAGs only.

Since the onset of angiogenesis is a key event in brain tumour progression, TAGs expressing angiogenic factors may display additional tumour-promoting effects compared to non-angiogenic TAGs. Thus, we also dissociated mature, vascular GBM xenografts and separated glioma cells and TAGs from the suspensions. Glioma cells were subsequently re-implanted in mice (*n* = 15) as pure glioma cell suspensions (*n* = 5) or as mixtures of glioma cells and either TAGs (*n* = 6) or unconditioned glial cells from normal mouse brains (*n* = 4, Fig. 2b, right panel). In addition, 4 mice received pure TAG populations. Mice co-implanted with glioma cells and TAGs had a median survival of 87 days, significantly shorter than for mice implanted with only glioma cells (median survival 103 days, *p* = 0.001) and for mice implanted with glioma and normal glial cells (median survival 101 days, *p* = 0.032). The slightly shorter survival for mice receiving normal glial and glioma cells compared to mice implanted with only glioma cells was not significant (*p* = 0.17). Mice receiving only TAGs remained well until the experiments were terminated after 6 months, when histopathological examination showed no tumour engraftment.

### Tumour-associated glial cells have a distinct gene expression profile and express markers associated with primitive glia and CAFs

**Fig. 3:**
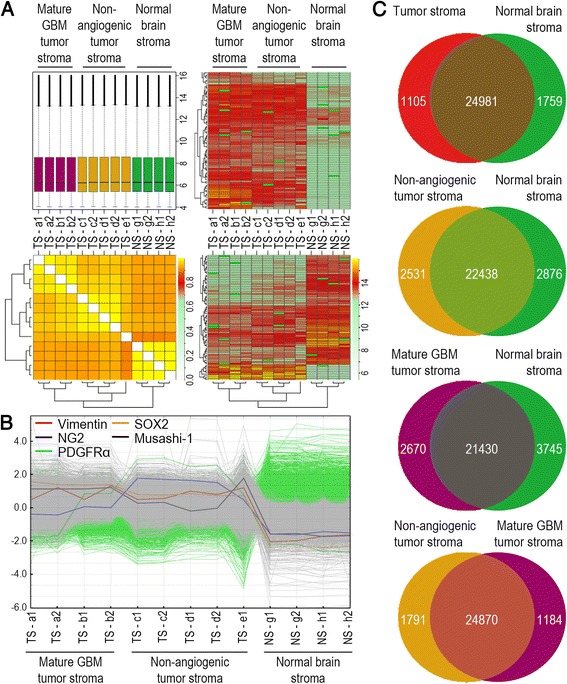
TAGs display a distinct gene expression profile. **a** Boxplot showing the normalised fluorescence intensities of the labelled samples that were analysed (*upper left panel*). Global hierarchical clustering of the samples (*lower left panel*) shows that samples from normal glial cells and TAGs from non-angiogenic and mature GBM phenotypes group separately. Global hierarchical clustering analysis over both genes and samples (*right panels*) show TAG gene clusters, both over- and underexpressed compared to normal glial cells. **b** Mean normalised expression profiles of “stemness” genes are upregulated in TAGs. **c** The numbers of significantly overexpressed genes in various groups are shown in their respective compartments in the non-overlapping areas (SAM analysis, q < 0.05). Genes that are not significantly differentially expressed are shown in the overlapping areas

We next performed gene expression profiling, comparing TAGs from non-angiogenic and mature GBM tumour phenotypes to normal glial cells using the Agilent Whole Mouse Genome 4 × 44 K Oligo Microarray. In total, we analysed nine TAG samples, including four samples from mature GBM and five samples from non-angiogenic tumour phenotypes, and four samples from healthy mice brains. Normalised fluorescence intensities showed little variation between the samples (Fig. 3a, upper left panel).

Global hierarchical clustering, grouping individual samples on the basis of similarity in gene expression with other cases, showed groupwise segregation of glial cells from normal brain and TAGs from the two tumour phenotypes. Thus, all samples displayed a higher within-group similarity, than with samples from other groups. We observed this pattern for all genes (Fig. 3a, lower left panel), and when analysis was restricted to significantly differentially expressed genes (q < 0.05) between TAGs and normal glial cells. Global hierarchical clustering analysis over both genes and samples showed patterns of over- and underexpressed genes in the TAG cell population, relative to glial cells from normal brain (Fig. 3a, upper and lower right panels). Among these, genes associated with stem cells and precursor phenotypes were upregulated in TAGs from both tumour phenotypes compared to normal glial cells (Fig. 3b). *Sox2*, a transcription factor associated with self-renewal [31], and *Musashi-1*, involved in asymmetric cell division [32], were both significantly upregulated in TAGs. In addition, the CNS-specific foetal transcription factor *Pou3f2*, and markers of neural stem cells and primitive glia, such as *Vimentin* and *Ng2*, were significantly upregulated. These latter markers have also previously been associated with CAFs [33]. *Pdgfrα,* which reportedly is expressed in pulmonary cancer-associated fibroblasts [34], was also upregulated in TAGs. In total, 2864 genes were differentially expressed between TAGs from all tumours and normal glial cells (Fig. 3c, upper panel). Of these, 1105 genes were overexpressed in TAGs, while 1759 genes were overexpressed in normal glial cells (Additional file 2: Table S1). Since TAGs were obtained from two different tumour phenotypes, we also compared TAGs from each phenotype separately with normal glial cells. 5407 genes were differentially expressed between normal glial cells and TAGs from the non-angiogenic glioma phenotype (Fig. 3c, second panel from top), while 6415 genes were differentially expressed between normal glial cells and TAGs from the mature GBM phenotype (Fig. 3c, second panel from bottom). Thus, more genes were differentially expressed when normal glial cells were compared to TAGs from the individual tumour phenotypes, as opposed to comparing with both phenotypes collectively. This may reflect that TAGs from the two tumour phenotypes are different and carry unique signatures that become masked when they are grouped. Therefore, we also compared TAGs between the two phenotypes, and found that 2975 genes were differentially expressed between TAGs derived from non-angiogenic tumours and mature GBM phenotypes (Fig. 3c, bottom panel).

**Fig. 4:**
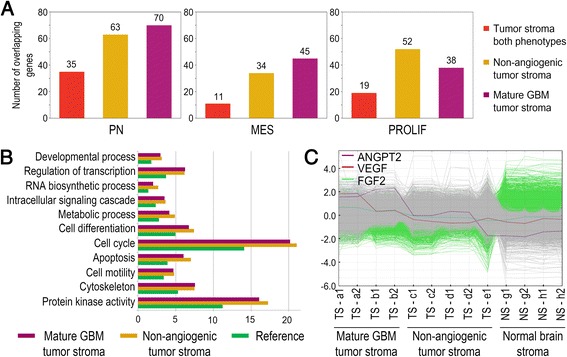
Gene ontology analysis of TAGs from non-angiogenic and mature GBM phenotypes. **a** Overlap between expression profiles of TAG populations as indicated and human subclasses of GBM. TAGs from both phenotypes combined displayed a significant overlap with both the proneural (*p* < 0.0001) and the proliferative (*p* < 0.0016) subclasses, but not the mesenchymal (*p* < 0.26) subgroup. Overlap between the individual TAG phenotypes and all the human GBM subclasses were highly significant (*p* < 0.0001). **b** The bar chart shows percentages of the genes ascribed to the biological processes indicated. All genes on the array chip were set as reference (*green*). The percentages of differentially expressed genes ascribed to these processes are significantly higher (Fisher’s exact test, *p* < 0.05 after Bonferroni correction) both in TAGs from non-angiogenic (*yellow*) and mature GBM phenotypes (*purple*). **c** Gene profiles of angiogenic factors significantly upregulated in TAGs from mature GBM phenotypes compared to TAGs from non-angiogenic tumour and normal glia. ANGPT2: Angiopoietin 2, VEGF: Vascular endothelial growth factor, FGF2: Fibroblast growth factor 2. PN: Proneural, MES: Mesenchymal, PROLIF: Proliferative

We next investigated whether TAG expression profiles had a resemblance to human expression profiles associated with prognostic subclasses of human gliomas [35] (Fig. 4a). Interestingly, analyses revealed that the TAG gene expression profiles were significantly enriched for genes contained within the human proneural, mesenchymal and proliferative glioma subgroups. Moreover, both TAGs from mature and non-angiogenic phenotypes displayed larger overlap with the proneural than any of the other human glioma subgroups.

Finally, gene ontology analysis revealed that differentially expressed genes were significantly enriched for gene clusters related to biological processes commonly altered in cancer, such as development, differentiation, cell cycle progression, apoptosis, cell motility and metabolism (Fig. 4b and Additional file 3: Table S2). Moreover, when comparing TAGs from the mature GBM phenotypes to TAGs from non-angiogenic gliomas, we observed a significant upregulation of *Vegf*, *Angiopoietin 2* and *Fgf2* (Fig. 4c).

### Glial host cells in human GBMs express TAG related markers

**Fig. 5:**
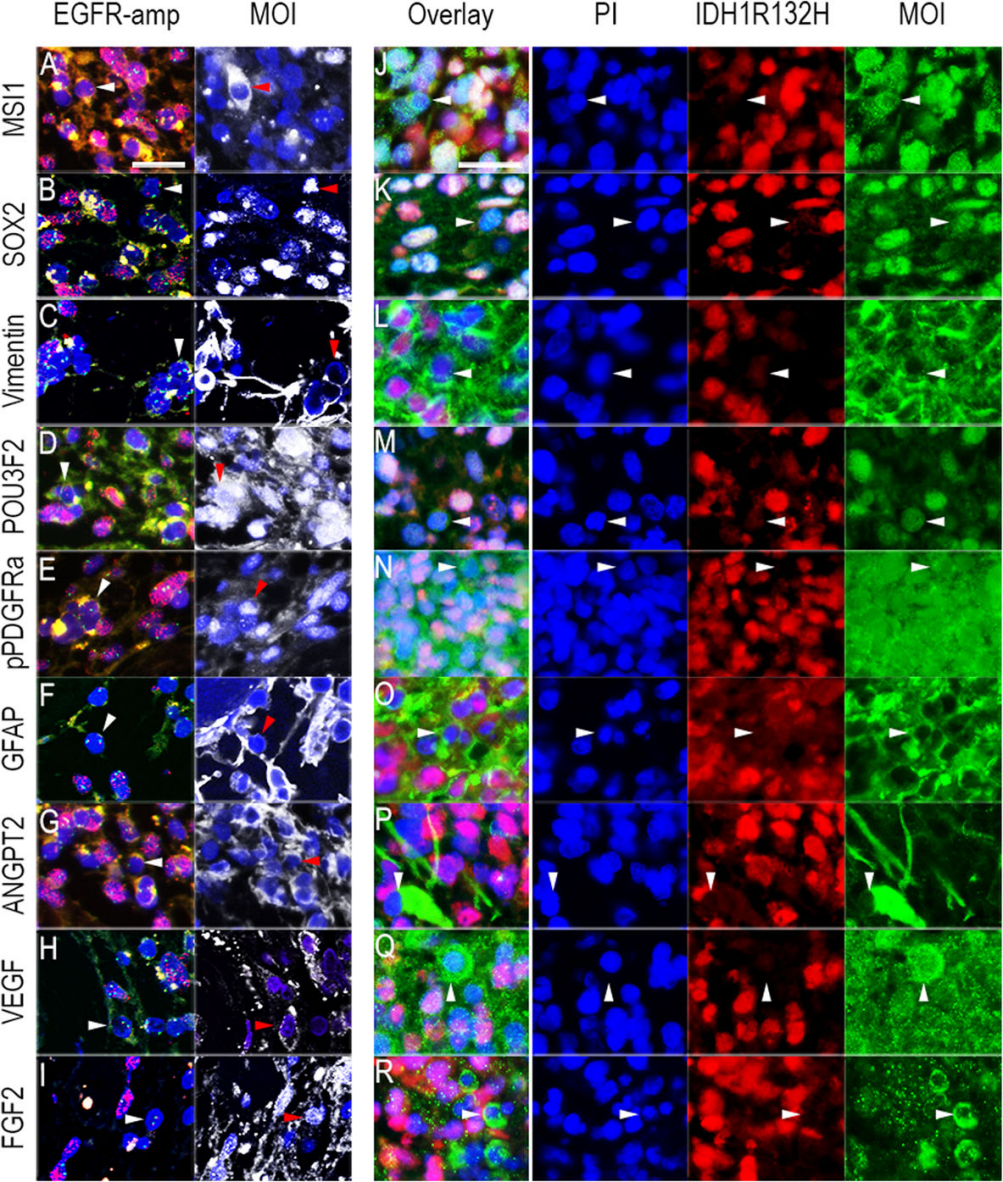
**a**-**i** EGFR (*red*) and CEP7 (*green*) FISH signals (*left panels*) and immunostaining of marker of interest (MOI, *white*, *right panels*). Images were taken from the same area on the same sample. *White* and *red arrowheads*: Cells with diploid of EGFR-CEP7 signals (two of each) that are positive for marker of interest, respectively. Counterstaining: Hoechst dye (*blue*). **j**-**r** Double staining for IDH1R132H-mutation (pseudored) and MOI (*green*). *White arrowheads*: Cells that are negative for IDH1R132H-mutation but positive for MOI. Counterstaining: PI (pseudoblue). Scale bars: 25 μm

The glial host cell expression of markers for immature cells, self-renewal and angiogenesis was confirmed with immunostaining of human GBMs. In order to identify host cells, we validated these markers in an EGFR amplified human GBM, combined with FISH (assessing EGFR chromosome gain and gene amplification) using EGFR probes and CEP7 probes for the centromere region of chromosome 7, (Fig. 5a–i). We also performed doublestaining of an IDH1 mutated GBM with antibodies both for the IDH1 mutation and the markers of interest (Fig. 5j–r). Notably, both samples showed stromal and tumour cell expressions of markers associated with self-renewal and differentiation of CNS cell types such as Musashi-1, SOX2, POU3F2, PDGFRA, GFAP and Vimentin, as well as angiogenic factors including FGF2, VEGF and Angiopoietin 2. However, GBMs display genetic heterogeneity. Snuderl et al. reported that 4–5% of GBMs expressed more than one receptor tyrosine kinase (RTK) [36]. Notably, the RTKs were not present in the same tumour cell, providing a mosaic amplification of RTKs within this subgroup of GBMs. Although this was a relatively rare phenomenon, it introduces the risk of mislabelling tumour cells as stromal cells when using tumour cell mutations as the defining criteria. We therefore conducted immunofluorescence doublestaining of a GBM xenograft staining against the pan-human specific nuclear antigen HuNu, to obtain a definite distinction between glioma (violet nuclei) and host cells (blue) in the tumour bed (Additional file 4: Figure S2a–t). Moreover, staining GBM xenografts allowed us to assess the spatial distribution of immunopositive cells throughout the tumour bed. Again, we observed that several factors were expressed both by tumour and host cells in the tumour core, including Musashi-1 (Additional file 4: Figure S2a, f) and SOX2 (Additional file 4: Figure S2b, g). Interestingly, host cell expressions of several markers were strongly upregulated towards the tumour bulk, but hardly visible outside the tumour region, including Vimentin and the angiogenic factors Angiopoietin 2, VEGF and FGF2. Similarly, PDGFRA expression, previously reported to mediate recruitment of stromal cells [34], was not detected in the parenchyma distant to the tumour, but abundantly expressed in host cells infiltrating the tumour (Additional file 4: Figure S2e, j).

## Discussion

Our limited knowledge about brain tumour-host interactions is mostly based on studies of tumour angiogenesis involving the host vasculature and brain tumour immunity. Recently however, several reports have implicated glial cells in this interplay [12, 14].

In this study, we isolated glial host cells from GBM xenografts established in GFP-NOD/scid mice. Combined with negative selection, by removing stromal endothelial (CD31^+^) and white blood (CD11b^+^) cells, we obtained a cell population expressing various glial cell markers but only low levels of the neuronal β-tubulin III. Notably 96% of these cells expressed GFAP confirming their glial phenotype. However, since glioma cells also express glial markers, we confirmed their host origin using three different approaches: 1) Control fluorescence microscopy for host cell GFP expression following every sorting, 2) Immunofluorescent staining confirming the absence of the pan-human specific marker Human Nuclear factor (HuNu) in the stromal compartment, and 3) By implanting GFP^+^ CD11b^−^ CD31^−^ cells in NOD/scid mice, which were non-tumourigenic compared to the implanted glioma cells.

Since immunofluorescence and FCM demonstrated that GFP^+^ CD11b^−^ CD31^−^ cells isolated from the GBM xenografts were highly enriched for various glial markers, we named them tumour-associated glial cells (TAGs). Had we instead isolated host cells from the tumour bed by positive selection, guided by predefined glial markers, cell populations expressing other glial markers would have been missed. Thus, we believe the observed expressions of several glial markers supports our unbiased approach with isolation of all host cells, followed by removal of cells expressing vascular and endothelial cell markers. In the TAG populations, the percentages of positive cells for the various markers exceeded 100%, indicating that TAGs express several glial markers simultaneously. However, the expression of multiple glial markers is also consistent with the presence of different subpopulations of glial host cells. Whether these subpopulations are also functionally distinct entities is not known. Future studies aimed at characterisation of glial cells grouped by marker expression may help clarify the role of different subpopulations. Notably, a significant proportion of TAGs were in the S/M-phase and TAGs also expressed Nestin. In addition, TAGs displayed a sphere forming ability in stem cell medium. The presence of a TAG subpopulation with stem-like properties may reflect a reprogramming of mature glial cells in the tumour bed, recruitment of neural precursor cells or bone marrow-derived cells, or a combination of these. Thus, the origin of TAGs is not established, and they may in principle have several different sources.

Moreover, TAGs displayed a gene expression profile distinct from glial cells from healthy brains, including an upregulation of growth factors and growth factor receptors consistent with an active role in the tumour-host interplay. Interestingly, this included markers also expressed by CAFs, suggesting that conditioning of glial cells in the brain tumour microenvironment resembles the activation of fibroblasts in other tumours [33, 34]. In addition, their sphere forming ability in stem cell medium was corroborated by an upregulation of immature markers and foetal transcription factors. Importantly, TAGs and glioma cells share a common environment and are exposed to the same external cues. Thus, the niche that endows glioma cells with stem-like properties may induce similar changes in neighbouring TAGs.

Furthermore, TAGs from non-angiogenic and vascular GBM xenografts had different expression profiles, suggesting that TAGs co-evolve with the tumour cell compartment during tumour progression. Notably, TAGs from the vascular GBM xenografts upregulated angiogenic factors. This is consistent with their tumour-promoting effects and may underlie the significantly shorter survival we observed in our co-implantation experiment with TAGs from vascular GBM xenografts. The difference in median survival from 103 days when implanting glioma cells only, to 87 days when co-implanting glioma cells and TAGs, may seem marginal but still provides proof-of-concept that brain tumour-stroma interactions may impact on survival. It is therefore conceivable that the brain tumour-host interplay can be targeted therapeutically.

Previously, our model has been shown to histopathologically closely mimic patient tumours. Importantly, the clinical validation of our gene expression data in human GBMs, both with IDH1 mutations and with EGFR amplifications, further confirms their relevance to a patient setting. A recent study however, showed that a fraction of GBMs contains genetically distinct subclones, with different tyrosine kinase mutations. Although this fraction was reportedly small, representing 4.5% of a cohort with 350 GBMs, it introduces the risk of misidentifying tumour cells without the IDH mutation/EGFR amplification as stromal cells [36]. We therefore conducted additional immunostaining of human GBM xenografts for the markers of interest combined with a human-specific antibody to obtain a definite identification of stromal cells. Apart from confirming the previously performed immunostainings on patient samples, it provided additional information by showing the spatial distribution of host cells expressing TAG-related genes. Interestingly, expression of upregulated genes in the TAG samples, were mostly confined to the tumour bed, whereas glial cells in the surrounding brain further away from the tumour expressed these proteins at much lower levels. We believe this further demonstrates that the TAG gene expression profile reflects a conditioning to the tumour microenvironment.

Previous genomic analyses of large GBM patient cohorts have shown that EGFR and IDH1 mutated GBMs fall in different prognostic GBM subclasses [37]. Since these analyses are based on patient biopsies containing both tumour and stromal cells, both cell compartments may in principle contribute to a tumour’s genetic profile. Previously, Katz et al. [15] investigated gene expressions in GFAP expressing host cells from murine glioma of heterozygously deleted *Ink4a/Arf* mice. Notably, their microarray analysis also identified genes associated with survival. Interestingly, we found that genes constituting the TAG gene expression profile were significantly overrepresented in all human GBM subclasses. Although the significance of these findings cannot be fully interpreted and understood from this study alone, it suggests that the reprogramming in glial cells of the tumour microenvironment has a clinical relevance. Future studies may clarify whether the gene expression of TAGs also provides a prognostic signature: i.e. whether TAG profiles from individual patient samples show higher similarities to the subgroup predicting the patient’s survival outcome.

## Conclusions

The present data demonstrate that TAGs have a distinct gene expression profile, involving stem cell and immature markers. Moreover, TAGs contain a significant proportion in S or G_2_/M-phase and display sphere formation in stem cell medium further suggesting a stem-like phenotype. Notably, TAGs from highly vascular xenografts also upregulate angiogenic factors and promote brain tumour growth in vivo. In summary, these findings establish a role for TAGSs as active participants in the brain tumour-host interplay. However, the mechanisms behind activation of glial cells in the tumour microenvironment are incompletely understood.

## Additional files

Additional file 1: Figure S1. MRI, H/E and flow cytometry of non-angiogenic and mature GBM phenotypes. A) Upper panels: MRI of non-angiogenic tumour. Contrast-enhanced T1 sequence (left) shows no enhancement. T2 (right) shows a diffuse lesion (red arrowhead). Lower panels: MRI of mature GBM phenotype. Contrasted T1 (left) shows enhancement (white arrowhead) due to leaky tumour vessels and dark areas of necrosis (red arrowhead). T2 (right) shows the tumour lesion. B) H/E staining of nonangiogenic (upper) and mature GBM tumours (lower). Non-angiogenic tumour with infiltrative growth (left) and cellular atypia (right). The mature GBM phenotype displays shift of midline structures (lower, left panel) and enlarged vessels, necrotic regions (white arrowhead) and pseudopalisading cells (red arrowhead) surrounded by dilated vessels (black arrowhead, lower right panel). Scale bars: left and right panels 100 μm. C) Scatter plot of the cell suspension: gating for live cells (left), gating by APC (CD11b and CD31) and GFP (right). D) Gating hierarchy and percentage distribution of different cells types. E) Percentages of TAGs in the two tumour phenotypes as indicated. TS: tumour sample, −a, −i etc. refers to the individual samples. F) Expression of various glial markers in GFP + CD11b-CD31- TAGs and GFP + CD11b-CD31- cells from normal mouse brains. (JPG 1941 kb)
Additional file 2: Table S1. Gene list of all significantly over- and underexpressed genes in pairwise comparisons, sorted after fold change value. (XLS 921 kb)
Additional file 3: Table S2. GO term list of all significant GO terms in pairwise comparisons, sorted after enrichment value. (XLS 127 kb)
Additional file 4: Figure S2. Expression of stem cell markers and angiogenic factors in GBM xenografs. A-T) Immunostaining against the markers (green) indicated in left panels. Sections are also stained for the pan human specific marker HuNu (red). Nuclear counterstaining: DAPI (blue). Left panels: Low magnification (×10 a, b and d, ×20 c, e and k-o) showing the tumour bulk and periphery. The tumour cell nuclei appear violet due to red HuNu and blue counterstaining. Right panels: High magnification (×80 f-j and p-t, and inserts y160 f-j and p-t) of the tumour bed. Scale bars: left panels 100 μm (a-e and k-o), right panels 25 μm (f-j and p-t) and inserts 5 μm (f-j and p-t). (JPG 3534 kb)

## Abbreviations

CAFs: Cancer-associated fibroblasts
CNS: Central nervous system
ECM: Extracellular matrix
EGFR: Epidermal growth factor receptor
GBM: Glioblastoma
GFAP: Glial fibrillary acidic protein
GFP: Green fluorescent protein
IDH1: Isocitrate dehydrogenase 1
MBP: Myelin basic protein
MR: Magnetic resonance
NOD/scid: Non-obese diabetic/severe combined immunodeficiency
PDFGR: Platelet derived growth factor receptor
RTK: Receptor tyrosine kinase
TAGs: Tumour-associated glial cells
