# Supplementary figures and images for: Tumour-associated glial host cells display a stem-like phenotype with a distinct gene expression profile and promote growth of GBM xenografts
Source: BMC Cancer. 2017 Feb 7;17:108. doi: 10.1186/s12885-017-3109-8 (PMC5294893; doi:10.1186/s12885-017-3109-8)

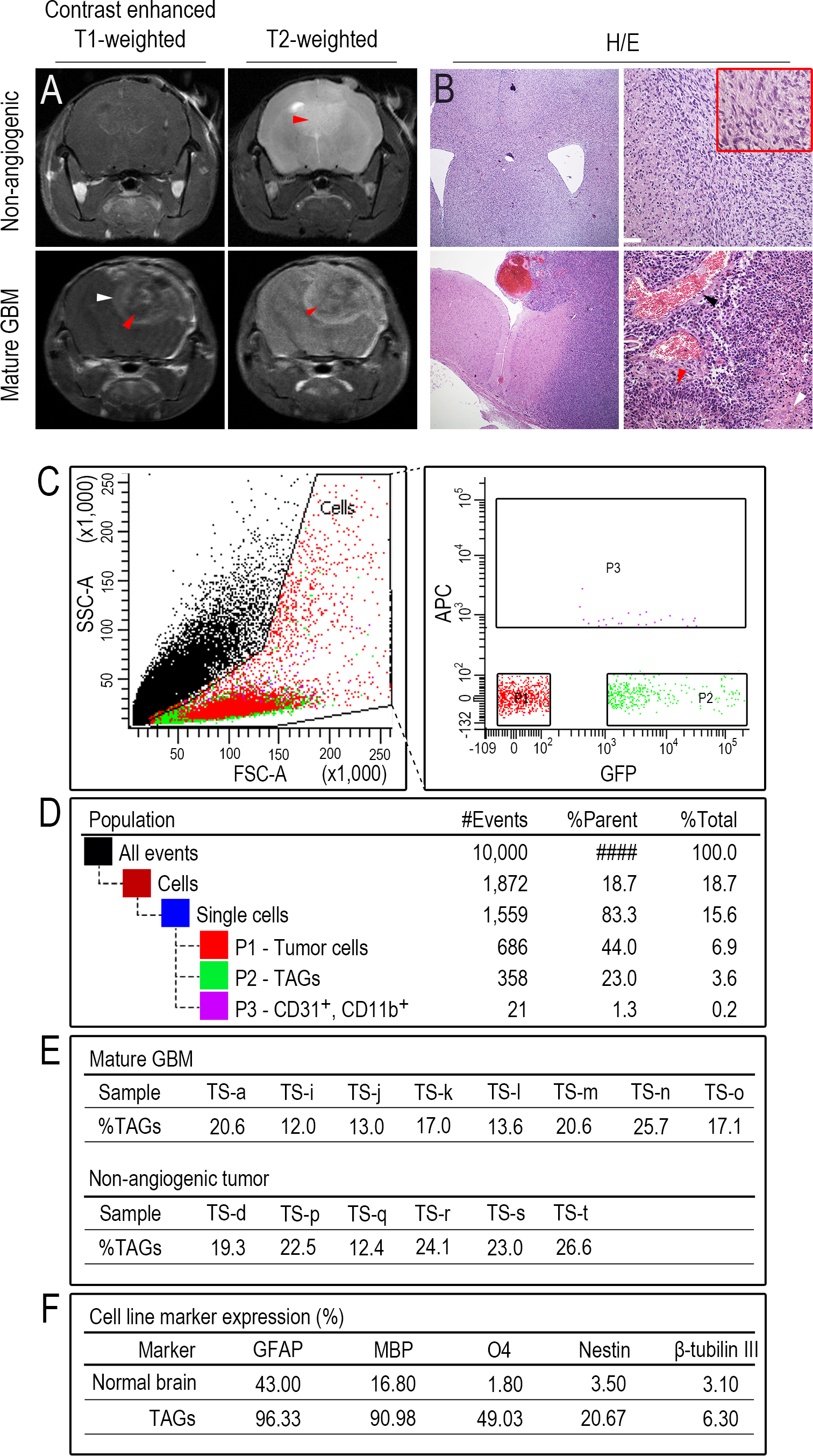

Supplement: Additional file 1: Figure S1. — MRI, H/E and flow cytometry of non-angiogenic and mature GBM phenotypes. A) Upper panels: MRI of non-angiogenic tumour. Contrast-enhanced T1 sequence (left) shows no enhancement. T2 (right) shows a diffuse lesion (red arrowhead). Lower panels: MRI of mature GBM phenotype. Contrasted T1 (left) shows enhancement (white arrowhead) due to leaky tumour vessels and dark areas of necrosis (red arrowhead). T2 (right) shows the tumour lesion. B) H/E staining of nonangiogenic (upper) and mature GBM tumours (lower). Non-angiogenic tumour with infiltrative growth (left) and cellular atypia (right). The mature GBM phenotype displays shift of midline structures (lower, left panel) and enlarged vessels, necrotic regions (white arrowhead) and pseudopalisading cells (red arrowhead) surrounded by dilated vessels (black arrowhead, lower right panel). Scale bars: left and right panels 100 μm. C) Scatter plot of the cell suspension: gating for live cells (left), gating by APC (CD11b and CD31) and GFP (right). D) Gating hierarchy and percentage distribution of different cells types. E) Percentages of TAGs in the two tumour phenotypes as indicated. TS: tumour sample, −a, −i etc. refers to the individual samples. F) Expression of various glial markers in GFP + CD11b-CD31- TAGs and GFP + CD11b-CD31- cells from normal mouse brains. (JPG 1941 kb) [file 12885_2017_3109_MOESM1_ESM.jpg]

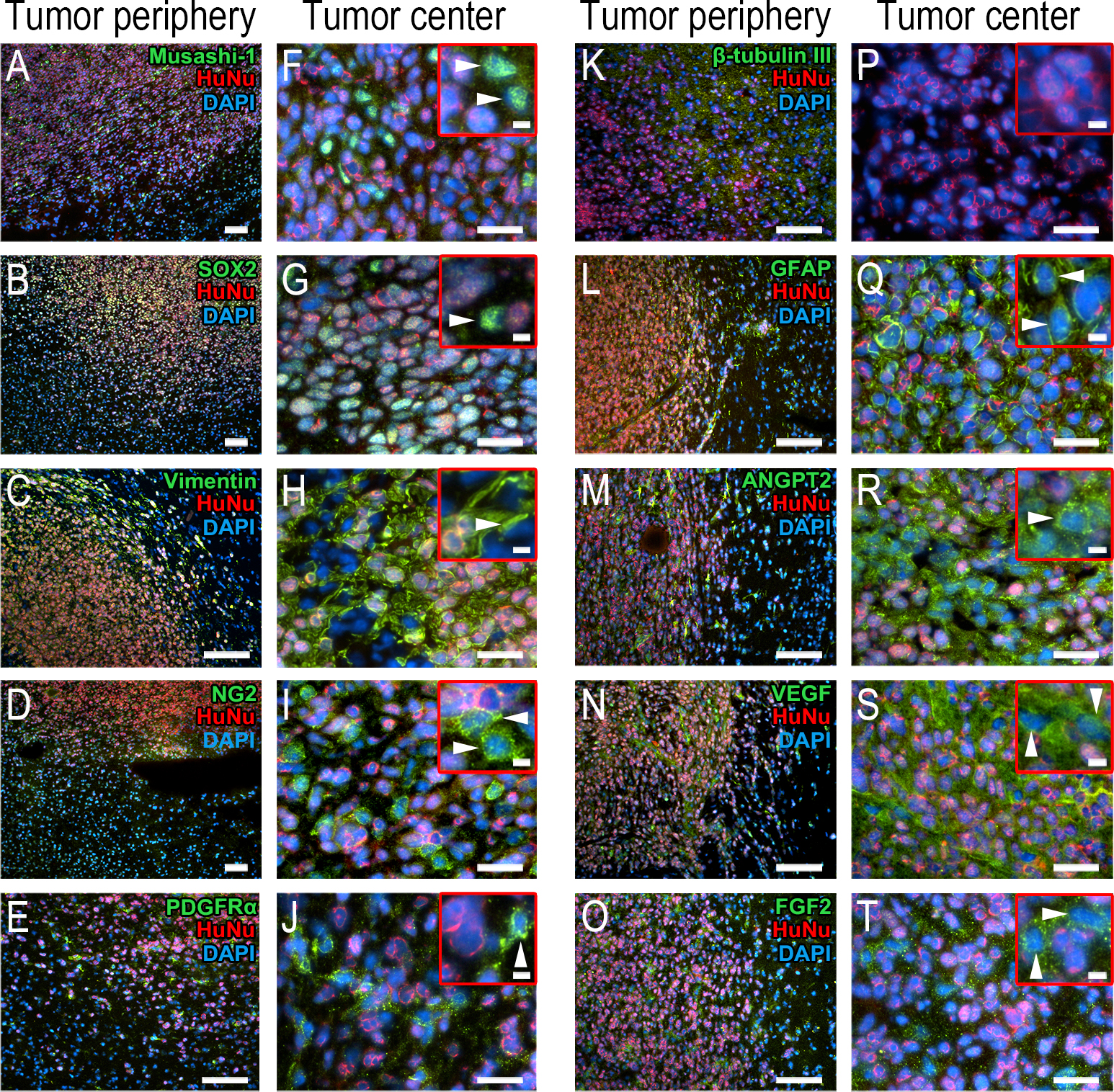

Supplement: Additional file 4: Figure S2. — Expression of stem cell markers and angiogenic factors in GBM xenografs. A-T) Immunostaining against the markers (green) indicated in left panels. Sections are also stained for the pan human specific marker HuNu (red). Nuclear counterstaining: DAPI (blue). Left panels: Low magnification (×10 a, b and d, ×20 c, e and k-o) showing the tumour bulk and periphery. The tumour cell nuclei appear violet due to red HuNu and blue counterstaining. Right panels: High magnification (×80 f-j and p-t, and inserts y160 f-j and p-t) of the tumour bed. Scale bars: left panels 100 μm (a-e and k-o), right panels 25 μm (f-j and p-t) and inserts 5 μm (f-j and p-t). (JPG 3534 kb) [file 12885_2017_3109_MOESM4_ESM.jpg]
